# Degree of exposure and peritraumatic dissociation as determinants of PTSD symptoms in the aftermath of the Ghislenghien gas explosion

**DOI:** 10.1186/s13690-015-0069-9

**Published:** 2015-04-13

**Authors:** Erik De Soir, Emmanuelle Zech, Ann Versporten, Herman Van Oyen, Rolf Kleber, Jacques Mylle, Onno van der Hart

**Affiliations:** Department of Scientific and Technological Research, Royal Higher Institute of Defence, Avenue de la Renaissance, 30 B-1000 Brussels, Belgium; Faculty of Pychology and Educational Sciences, Research Center for Health and Psychological Development, Université catholique de Louvain, Place du Cardinal Mercier 10, B-1348 Louvain la Neuve, Belgium; Vaccine & Infectuous Disease Institute (VAXINFECTIO), Faculty of Medicine and Health Science, Laboratory of Medical Microbiology, University of Antwerp, Universiteitsplein 1, B-2630 Antwerp, Belgium; Scientific Institute of Public Health, Direction Public Health and Surveillance, J. Wytsmanstreet 14, B-1050 Brussels, Belgium; Department of Clinical and Health Psychology/Arq Psychotrauma Expert Group, Faculty of Social Sciences, Utrecht University, Utrecht/Diemen, The Netherlands; Department of Behavioral Sciences, Royal Military Academy, Avenue de la Renaissance 30, B-1000 Brussels, Belgium; Department of Clinical and Health Psychology, Faculty of Social Sciences, Utrecht University, Utrecht, The Netherlands

**Keywords:** Technological disaster, PTSD symptoms, Peritraumatic dissociation, Social support, Psychological help

## Abstract

**Background:**

This paper investigates risk factors for the development of posttraumatic stress symptoms in the different survivor groups involved in a technological disaster in Ghislenghien (Belgium). A gas explosion instantly killed five firefighters, one police officer and 18 other people. Moreover, 132 people were wounded among which many suffered severe burn injuries.

**Methods:**

In the framework of a large health survey of people potentially involved in the disaster, data were collected from 3,448 households, of which 7,148 persons aged 15 years and older, at 5 months (T1) and at 14 months (T2) after the explosion. Hierarchical regression was used to determine the significant predictors and to assess their proportion in variance accounted for.

**Results:**

The degree of exposure to the disaster was a predictor of the severity of posttraumatic stress symptoms. Peritraumatic dissociation appeared to be the most important predictor of the development of posttraumatic stress symptoms at T1. But at T2, posttraumatic stress symptoms at T1 had become the most important predictor. Dissatisfaction with social support was positively linked to development of posttraumatic stress symptoms at T1 and to the maintenance of these symptoms at T2. Survivors who received psychological help reported significant benefits.

**Conclusions:**

In harmony with the findings from studies on technological disasters, at T1 6,0% of the respondents showed sufficient symptoms to meet all criteria for a full PTSD. At T2, 6,6% still suffered from posttraumatic stress symptoms. The symptoms of the different victim categories clearly indicated the influence of the degree of exposure on the development of posttraumatic stress symptoms. Problems inherent to retrospective scientific research after a disaster are discussed.

## Background

In Western countries, 5% of all deaths are due to the consequences of aggressive or unnatural, technological, events [1]. These events may cause posttraumatic stress symptoms [2,3]. When these symptoms lead to such a severe suffering that they hamper social, familial or professional functioning for more than one month, they are indicative of PTSD [4]. Studies indicated that the prevalence of PTSD during the first year after a disaster is 1 to 11% [1,5-7]. Disaster victims are often difficult to define and the denominator is usually unclear in the direct aftermath of disaster. For this reason, disaster research often focuses on the residents of the official disaster area [8] or survivors who had to be relocated after the disaster [9]. In other cases [10], victims are identified through the medical chain and as a function of their medical condition. The posttraumatic consequences of disasters (i.e., the percentage of survivors with PTSD) depend on the type of exposure to it. For instance, two months after accidents with serious injuries 35% of survivors met PTSD criteria [11,12], five to eight weeks after the disaster depending on the proximity to the World Trade Center site 7 to 20% respondents met the PTSD criteria [13], and ten years after a disaster on an oil platform 25% continued to satisfy PTSD criteria [14]. This indicates that there exists a relationship between the severity of exposure and the mental health condition, called dose-relationship, which could serve as a basis for victims classification. Severe exposure such as threat of life, the confrontation with injury and human losses or severe initial stress reactions may be considered as event-specific risk factors [15,16].

In Belgium, the impact of two major accidents [2,3] has been investigated: (1) a sudden fire blaze in a ballroom in which about 450 guests were celebrating the 1994 New Year Eve (Switel Hotel, Antwerp, Belgium) with 13 people killed and more than 120 with burn injuries; and (2) a massive car pile-up in 1996 (Nazareth, Belgium) involving about 150 cars and trucks, in which 10 people died and 50 others were injured. The Composite International Diagnostic Interview (CIDI) was used between 7 and 9 month after these potentially traumatizing events in a study group of 185 victims; 130 fire and 55 car accident victims. The incidence of PTSD for these accidents appeared to be 26.2% for the ballroom fire blaze and 16.4% for the massive car pile-up.

The varying degrees of PTSD in disaster survivors show that scientific research is not univocal with regard to the determinants of PTSD. Many studies showed methodological shortcomings, which might be inherent to research on the impact of disasters on (mental) health: the suddenness of a disaster, the lack of control groups [16], the different definitions of the concepts used (regarding the type of traumatizing event, psychological disturbances, dissociation, etc.) or the victim categories [17-21]. In the past few years, much importance has been attributed to the study of predictive factors concerning the development of psychological disturbances, such as the role of several phenomena occurring during or immediately after the potentially traumatizing impact. Reactions experienced immediately at or after the moment of the event, such as peritraumatic dissociation, extreme anxiety, panic, and negative emotions, appear to be important predictors of PTSD symptoms [22]. Symptoms of peritraumatic dissociation are thought to involve cognitive alterations and alterations of perceptual functioning at the moment of, or directly after the event [23,24]. A comprehensive meta-analysis [25] revealed that peritraumatic dissociation turned out to be the best PTSD predictor in comparison with other predictors such as earlier traumatization, earlier psychological well-being, familial antecedents of psychopathology, life threat felt during the traumatic event, social support and emotional reactions occurring during or right after the event (i.e. negative peritraumatic emotions such as guilt or self-blame).

Since disasters always strike networks of people, it is also important to investigate the effect of social support. Previous research [17,25] found that social support counters the development and maintenance of distress and mental disorders, in particular PTSD. Social sharing occurs in 80 to 95% of all emotional episodes and usually develops in the period immediately following an emotional event [26,27]. Modally, the sharing of an emotional episode is repetitive and addresses several successive recipients. Experimental studies confirmed emotional exposure to cause the social sharing of emotion [28]. Emotion sharing was consistently found to hold a positive linear relationship with the intensity of the emotional experience [28,29].

The current study aimed at 1) assessing the prevalence of posttraumatic stress symptoms in survivors of the Ghislenghien gas explosion, in their family members as well as in family members of deceased victims, 2) focusing particularly on six predictors of posttraumatic stress symptoms: the frequency and the severity of posttraumatic stress symptoms, the type of exposure, the level of peritraumatic dissociation, the received social support and the professional help.

On July 30th 2004, a leakage caused by a drilling machine in a high pressure gas pipe, which passed under the industrial zone of Ghislenghien (Belgium) created a persistent gas smell and alerted the employees of one of the factories. When the fire services arrived on-scene, an enormous explosion took place and instantly killed 24 people. Only two firefighters from the first crew survived the initial blast and 132 people were wounded. An impressive column of fire rose into the air and the heat was felt up to two kilometers away from the explosion site. Debris from the gas pipe and buildings was projected up to six kilometers away from the epicenter; up to 16 km from the explosion, air vibrations were registered. A wide area was affected by the largest technological disaster that Belgium ever knew since the mine disaster of Marcinelles (1956) in which 256 coal miners were killed by an underground fire.

## Methods

### Design of the study

In the framework of a health survey of people involved in the disaster, inhabitants living maximum five kilometers away from the explosion epicentre and employees of all companies located on the industrial site of Ghislenghien were contacted. The target group comprised 3,448 households, totaling 7,148 persons aged 15 years and older on August 1, 2004. All of them, as well as all their family members living at the same address and the family members of all deceased persons were invited to participate in the study. Fire, police, and emergency medical services personnel who took part in the rescue operations were excluded from the survey as they were the target of a specific investigation. Participation was voluntary. Questionnaires were sent by regular mail 5-months after the explosion (T1). Injured persons who did not react within one and a half months were contacted by telephone by experienced psychologists. Fourteen months after the disaster (T2), each person who responded to the first questionnaire received a second questionnaire for the follow-up of the impact of the disaster. To guarantee anonymity of the respondents and respect of the law on protection of private life, each questionnaire was given a unique, anonymous identification number.

### Measures

#### Degree of exposure

Twenty-three questions (yes-no) assessed how respondents had been involved in the disaster, allowing to elaborate a classification of the degree of exposure. This classification contained 3 main categories divided into 9 subcategories. The first category comprises the primary victims; i.e. persons who had been directly exposed to the disaster and were direct witnesses of human damage. Primary victims were subdivided in 5 subcategories: 1) injured and hospitalized for more than 72 hours; 2) injured and hospitalized less than 72 hours; 3) injured but not hospitalized; 4) not injured but were direct witnesses of human damage (injured or deceased); and 5) direct witnesses not exposed to human damage, i.e. direct witnesses of the explosion effects (heat, blast, etc.). The second category encompasses the secondary, indirect victims; i.e. who had been indirectly exposed to the disaster by being related to a primary victim. It includes family members or colleagues, either 6) of people who got injured or killed by the disaster, or 7) people who could have been injured or killed by the disaster but were for some reason not present on site). The third category regroups the tertiary victims; i.e. people classified as 8) could have been exposed directly to the disaster but were not because (accidentally not present on site, or 9) not belonging to any of the previous categories.

This classification constitutes an ordinal scale based on a “dose” dimension. The first category has been exposed to the highest dose of (life threatening) stress and category 9 to the least dosage. To a certain extent we can consider this scale as a continuous scale “compressed” into 9 ordered categories [30].

#### Peritraumatic dissociation

Peritraumatic dissociation was measured using the Peritraumatic Dissociative Experiences Questionnaire (PDEQ; [31]). Participants rated on a 5-point Likert scale the extent to which they respectively endorsed the 10 PDEQ-statements regarding their reactions during and immediately after the event. Since internal consistency was adequate (α = .85), an index of psychoform peritraumatic dissociation was computed by summing the item scores. The higher the total score, the higher peritraumatic dissociation.

#### Posttraumatic stress symptoms

The design of the study did not allow for PTSD diagnosis by clinicians. Therefore, an assessment of posttraumatic stress symptoms with the QE-PTSD, a French questionnaire, developed at the Université Catholique de Louvain and closely matching the DSM-IV criteria for diagnosing PTSD symptoms was used [32]. Criterion A1 (objective exposure to a potentially traumatic event) was assessed by questions on the type of exposure to the disaster described above. Three dichotomous questions (yes-no) addressed Criterion A2 (subjective exposure to the potentially traumatic event: intense fear, helplessness, horror). Criterion B (intrusions) was assessed by 8 items, and Criterion C (avoidance and numbing) and D (hyperarousal) by 7 items each. All these items had to be rated by the respondent on 5-point Likert scales (0 = not at all; 4 = a great deal). The threshold for a response to be considered positive was 2. Four items addressed Criterion F (trauma-related distress and impairment in social life, work, or daily activities (yes-no). Finally, three items evaluated (1) whether symptoms had lasted for more than a month (yes-no; Criterion E), (2) whether symptoms were still apparent (yes-no), and (3) when symptoms had started (month and year). Three types of variables were calculated: (1) a dichotomic index (satisfies vs not) for each PTSD criterion (showing 1 symptom for criterion B, 3 for C, and 2 for D) plus a PTSD index (criteria A-F satisfied or not), (2) a continuous variable of PTSD severity (sum of items scores B, C, and D endorsed), and (3) continuous variables representing the number of criteria met for each cluster of symptoms (B, C, and D). This QE-PTSD instrument had been tested on various samples of respondents after they had been exposed to a potentially traumatizing event [32-35]. The B, C and D criteria scales, as well as the total PTSD severity index, showed good internal consistency, respectively Cronbach’s alpha = 87, .76, .86, and .93 in Mutter’s sample [33], and .90, .83, .91, and .91 in the present sample.

#### Social sharing, social support, and professional support

The three dimensions usually addressed in social sharing of a given emotional event have been used in our study: latency, extent (number of repetitions), and number and type of targets [27,28]. Latency of the first sharing, extent of sharing and number of targets with whom they had shared was rated on a 7-points scale. The next question (yes/no) addresses the professionally provided support: Did you receive psychological help (personally or by telephone) after the event? Finally, a question focused on the support the involved persons offered to themselves to other victims: Did you provide support for other people involved in the disaster?

## Results

### Participation rates

At T1, the response rate at household level was 607 (18%) and at individual level, valid data were obtained from 1,027 adults (14%). At T2, valid data were obtained from 579 persons belonging to 338 households (46%). None of the family members of the 24 deceased persons participated in the study. Average age of the respondents was 45 years (SD = 16.6; range 15–92) and half of the sample (49.8%) were men. Nine percent of the participants (N = 95) at T1 were full time students. Among the remaining participants, 35.0% had completed higher education, 35.4% higher secondary education, and 29.5% had not finished secondary education.

### PTSD symptoms

For the assessment of PTSD symptoms, only people with complete data for at least one of the criteria (A1, A2, B, C, D, E or F) were taken into account. Some 1,4% of the respondents did not meet Criterion A1 because they were not at all involved in the disaster (e.g., being on holiday) (Table 1). They were excluded from further analysis. About 91% of the respondents satisfied criterion A2. All respondents (100%) satisfied criterion B and there was no significant decrease over time since all participants were still satisfying it at T2. Criterion C was satisfied by 9% of the respondents at T1 and the prevalence was 8% at T2. Nevertheless, 51% of those who satisfied this criterion at T1 (n = 23) did not so at T2 while 4% of the respondents (n = 20) had developed this type of symptoms at T2. The proportion of recovery at T2 was thus higher than the proportion of development at T2, χ^2^(1, N = 521) = 119.78, p < .0001. It should also be noted that some items, such as the efforts to avoid thinking about the event, were quite frequently endorsed (20%). Criterion D was satisfied by one third of the respondents (34%) at T1 and the prevalence stayed similar at T2 (33%). Nevertheless, 31% of those who showed hyperarousal symptoms at T1 (n = 60) did not show them anymore at T2, while 13% (n = 44) had developed this type of symptoms at T2. The proportion of persons who recovered from hyperarousal at T2 was thus higher than the proportion of persons who developed hyperarousal at T2, χ^2^(1, N = 527) = 169.72, p < .0001. It is worth noting that all respondents reported being more on edge and more watchful since the disaster. Criterion E (onset and duration) was satisfied by 52% of the participants at T1 and 53% at T2. Here, probably due to the longer time frame, the proportion of people who stopped satisfying this criterion at T2 (25%) was smaller than the proportion of who started endorsing this criterion at T2 (33%), χ^2^(1, N = 503) = 88.15, p < .0001. Finally, criterion F was satisfied by 26% of the respondents at T1 and 18% at T2. The proportion of persons who recovered at T2 was higher (57%, n = 81) than the proportion of new onsets of dysfunction at T2 (9%, n = 33), χ^2^(1, *N* = 510) = 78.74, *p* < .0001.

**Table 1:** **PTSD criteria satisfied after the Ghislenghien disaster (DSM IV, 1994)**

	**T1 : After 5 months**		**T2 : After 14 months**		**p-value χ-test**
	**N = 1027**	**% valid**	**N = 579**	**% valid**	
**Criterion A1 : objective exposure**	**1012**	**98.6**	**541**	**98.9**	
**Criterion A2 : subjective exposure**	**897**	**91.3%**	**485**	**92.2**	
Intense fear	503	56.8%			
Powerlessness	745	81.0%			
Horror	683	74.9%			
**Criterion B : Intrusions**	**978**	**99.9%**	**571**	**99.8%**	ns
**Criterion C : Avoidance**	**87**	**9.0%**	**48**	**8.4%**	.0001
**Criterion D : Hyperactivation**	**340**	**34.5%**	**189**	**32.9%**	.0001
**Criterion E : Duration of the disturbance (symptoms in B, C, D)**					
Duration of symptoms > 1 month	503	51.9	303	52.6%	.0001
Current presence of symptoms	350	36.6	219	38.0%	.0001
**Criterion F : Dysfunctioning**	**251**	**25.9%**	**103**	**18.3%**	.0001
**Current full PTSD pattern**	**54**	**6.0%**	**32**	**6.6%**	
**Resolved PTSD pattern at T1**	**6**	**.7%**			
**Resolved PTSD pattern at T2**	**--**	**--**	**14**	**3.0%**	
**Delayed PTSD pattern after T1**	**--**	**--**	**16**	**3.7%**	
**No PTSD pattern**			**444**	**90.6%**	

A total PTSD prevalence of 6% (n = 54) was found at T1, and of 6.6% (n = 32) at T2. The proportion of victims who did not show the symptoms required for a full PTSD anymore at T2 (51.9%, n = 14) was higher than the proportion of delayed onset of symptoms required for a full PTSD at T2 (4.1%, n = 18), χ^2^(1, *N* = 463) = 78.86, *p* < .0001.

### Determinants

#### Degree of exposure

Most of the 54 respondents satisfying all PTSD criteria at T1, belonged to the primary victims category; i.e. 40 were direct victims or direct witnesses of the disaster; 14 were burnt due to the explosion (14/31, 45% PTSD prevalence^a^), 7 had witnessed wounded or dead victims (7/48, 15% PTSD prevalence), and 19 had witnessed the explosion but not human damage (19/532, 4% PTSD prevalence). The most afflicted indirect victims of the disaster were the family members or colleagues of someone who was on the site (11/102, 11% PTSD prevalence). Finally, 3 persons that could have been on the site but were not had also developed PTSD symptoms (3/54, 2% PTSD prevalence). None of the proxies of persons that could have been on the site met all criteria for PTSD. Prevalence and severity of the symptoms index between the primary and the secondary victims at T1, were not significantly different, χ^2^(1, *N* = 510) = 2.46, *p* = .12 for PTSD prevalence and t < 1 for PTSD severity.

These results were confirmed by a repeated measure ANOVA examining the severity of the PTSD symptoms in the different exposure groups over time. The effect of time on the mean PTSD symptoms severity was not significant, *F*(1, 521) = 1.91, *p* = .17, η^2^ = .00, but the main effect of type of exposure was very significant, *F*(8, 521) = 16.26, *p* = .000, η^2^ = .20. Post-hoc analyses showed that the severity of the PTSD symptoms evolved in a significant way as follows; injured and hospitalized victims showed more severity than injured but not hospitalized victims who, in their turn, showed the same symptoms severity as the witnesses of human suffering. However, the latter witnesses showed more severity than those who witnessed the explosion only, sharing the same severity in symptoms with individuals who were close to direct victims of the explosion. Individuals who could have been involved in a direct or indirect way but have not been, showed still less severity in symptoms and shared the same level of severity as other non-victims. The interaction between time and the type of exposure tended to be significant, *F*(1, 521) = 1.67, *p* = .10, η^2^ = .03, which means that the intensity of the symptoms tended to evolve over time differently depending on the exposure group. Further analyses indicated that the impact decreased significantly for the witnesses of the explosion (*p* = .0001). A tendency toward significance was also found for the injured people hospitalized for more than 72 hours (*p* = .08), indicating a tendency for a decrease in PTSD severity from T1 to T2 and also for the people having someone close to them that could have been involved but was not (*p* = .10, same direction). For the other groups, intensity stayed stable over time.

#### Determinants of PTSD symptoms at T1 and T2

Potential predictors at T1 were entered in a hierarchical regression model. First, demographic variables (age and sex) were entered. Thereafter were entered successively the degree of exposure; peritraumatic dissociation; social support variables; and, finally, whether or not the person had received psychological help following the disaster. Results indicated that the severity of PTSD symptoms was strongly and positively related to type of exposure, peritraumatic dissociation, and dissatisfaction with social support (Table 2). However, the social support network, i.e., the number of friends or relatives on who people can count on in case of difficulties, was not related to the severity of symptoms and Age was neither. Finally, psychological help received after the disaster was negatively associated with symptoms severity.

**Table 2:** **Predictors of PTSD severity at 5 months (N = 700)**

	**β**	***t***	**Equation**	**Adj. R** ^**2**^	**ΔR** ^**2**^
*Sociodemographic variables*			*F*(2, 697) = 2.452^†^	.004	.007
1. Family belonging					
2. Age	.006	.208			
3. Gender	.044	1.654†			
*Type of exposure*			*F*(3, 696) = 49.491***	.172	.169
3. Exposure category	-.160	−5.483***			
*Peritraumatic dissociation*			*F*(4, 695) = 132.209***	.429	.256
4. Peritraumatic dissociation	.491	17.273***			
*Variables of social support*			*F*(7, 692) = 93.774***	.482	.055
5. Unsatisfaction with social support	.167	6.235***			
6. Number of supporting persons	-.038	−1.390			
7. Being an aid for other victims	-.127	−4.697***			
*Psychological support*			*F*(8, 691) = 97.034***	.524	.042
8. Received psychological support	-.223	−7.874***			

^†^ *p* < 0,10; *p < 0,05; **p < 0,01; ***p < 0,001.

A second hierarchical regression was performed for the prediction of PTSD symptoms severity at T2 (Table 3). In this analysis, in addition to the predictors at T1, the severity of PTSD symptoms at T1 was entered too. Results revealed that only two predictors remained significant at T2. First, the higher the intensity of the symptoms present at T1, the more respondents still presented these symptoms at T2. Second, the initial dissatisfaction with the social support remained positively related to the severity of the symptoms present at T2. Thus, neither the degree of exposure, nor the peritraumatic dissociation reactions predicted the severity of the symptoms at T2. Also, the psychological help initially received did no longer negatively determine the intensity of PTSD symptoms at T2.

**Table 3:** **Predictors of PTSD severity at 14 months (N = 384)**

	**β**	***t***	**Equation**	**Adj. R** ^**2**^	**Δ R** ^**2**^
*Sociodemographic variables*			*F*(2, 381) = 2.359^†^	.007	.012
1. Age	.046	1.570			
2. Gender	.006	.207			
*Type of exposure*			*F*(3, 380) = 20.764***	.134	.129
3. Exposure category	-.041	−1.210			
*Peritraumatic dissociation*			*F*(4, 379) = 40.778***	.294	.160
4. Peritraumatic dissociation	-.014	-.363			
*PTSD severity at 5 months*			*F*(5, 378) = 160.058***	.675	.378
5. Severity of PTSD symptoms at T1	.772	18.896***			
*Variables of social support at 5 months*			*F*(8, 375) = 102.430***	.679	.007
6. Unsatisfaction with social support	.073	2.399*			
7. Number of supporting persons	-.011	-.362			
8. Being an aid for other victims	-.042	−1.366			
*Psychological support*			*F*(9, 374) = 91.368***	.680	.001
9. Received psychological support	-.041	−1.260			

^†^p < 0,10; *p < 0,05; **p < 0,01; ***p < 0,001.

## Discussion

The main findings on the prevalence of PTSD symptoms are twofold. At T1, 6,0% of the respondents met all criteria for an indication of a full PTSD while this prevalence became 6,6% at T2. These results are in harmony with the findings from current literature [1,5,6] but at the same time is surprising that the prevalence stays the same over time. Striking is that almost every respondent reported mental intrusions of the disaster both at T1 and T2.

The results on the recovery of 51.9% of the victims that presented a full PTSD image at T1 and did not present it anymore at T2 are in accordance with research findings [36] that indicate how a majority of victims of disaster may react in a resilient way in the aftermath of extremely stressful experiences. The late-onset of PTSD symptoms at T2 is in accordance with the results of a recent study on the occurrence of mental health problems in the immediate aftermath of a fireworks disaster in Enschede [37] in which 4% of the participants demonstrated late-onset PTSD. They reported high initial intrusion and avoidance and experienced progression of these symptoms. They were more likely than all other participants to use mental health services several years after the disaster. In this study, severe disaster exposure and perceived lack of social support were associated with late-onset of symptoms. This study confirms these results.

The results also show that several risk factors are associated with the severity of the PTSD symptoms. Demographic variables, particularly age and gender, did not appear to have predictive value. This is contrary to previous findings of studies [7,36] which might have focused more on the impact of interpersonal trauma such as rape or assault instead of disaster.

Concerning the kind of exposure, the degree of potential life threat turned out to be an important determinant of the symptoms severity. Having been a direct witness of human damage or an indirect witness - being close to someone who died or got injured during the explosion - is a risk factor to a lesser extent.

The PTSD symptoms of the different victim categories showed the influence of the degree of exposure. In fact, 56 to 67% of the people who were injured and hospitalized presented a PTSD pattern at T1 and/or T2. Of the respondents who were directly exposed to the victims, either at the moment of the explosion, or because they were close to direct victims, 22 to 31% presented a PTSD symptoms pattern at T1 and/or T2. Finally, 99 to 100% of the respondents who could have been involved but were not exposed directly to the explosion or by the intermediate of someone who is close to them, never presented all the symptoms required for a full PTSD. These results indicate that the prevalence of PTSD symptoms developed as a function of the degree of exposure to the disaster and that this variable may serve as an important predictor of post-disaster PTSD symptoms. However, it remains less clear whether the type of victim, i.e., primary vs. secondary victims, would also be a relevant predictor of PTSD. Thus, it is necessary to examine more thoroughly the kind of exposure to the disaster.

The third factor, peritraumatic dissociation, was the most powerful PTSD predictor at T1, but the results of the second regression model showed that it had no longer the same predictive value at T2. A PTSD pattern at T1 appeared to be the biggest predictor of a PTSD pattern at T2. However, the absence of the evaluation of possible confounding variables in the relationship between peritraumatic dissociation and posttraumatic stress symptoms, as discussed in other studies [38], should caution against the view of peritraumatic dissociation as an independent predictor. For instance, controlling for pre-existing mental health problems could modify the current insights about peritraumatic dissociation. This is in line with other findings, which suggest that the reports of peritraumatic dissociation during or immediately after the particular event may be biased by the current psychological state of the affected individual [39-42]. Previous research [43] indicates that the primary risk for PTSD is less whether one dissociates during the traumatic events, than whether such dissociation persists over time. Whereas peritraumatic dissociation ceases to predict PTSD at the multivariate level [44] trauma-related persistent dissociation is a substantial predictor of PTSD. This study did not offer the possibility of gathering data on the pre-disaster mental health of participants or on persistent and generalized dissociation which continue to relate to PTSD status on the long term. It is unclear whether or not these findings are also applicable to the Ghislenghien disaster victims. Nevertheless, it is still advisable to include peritraumatic dissociation in screening measures intended to identify victims at risk for chronification of posttraumatic sequelae.

The development of PTSD symptoms was positively related to dissatisfaction with the provided social support, but not with the number of people (potentially) providing support which is in line with previous research [45,46]. Psychological help received after the disaster was associated with development of PTSD symptoms. It is not possible to provide an unambiguous interpretation for these findings because it might be that psychological help has not been useful or that this kind of help has been provided to those in the biggest need. But the latter hypothesis is not really confirmed by the results since only 60% of the respondents who developed PTSD symptoms (N = 31) have received psychological help and some of those who developed PTSD symptoms (N = 21) did not receive help while seven of these respondents declared that they had been in need for help.

### Strengths and limitations

One of the positive aspects of this study is its specificity, adding knowledge to the consequences of technological or man-made disasters in which a massive explosion causes human suffering. The longitudinal character of the Ghislenghien study allows to shed another light on the predictors of mental health disturbances based on an original classification of disaster survivors. The specific instruments developed for this study may enhance systematic and comparative research on the health consequences of technological disasters in the future. However, the results also reflect the problems inherent to scientific research after a disaster. As always, there is the suddenness of the disaster and the difficulty in defining a control population. In this case, there was also a delay due to the time needed to the elaboration of partnerships, research protocols/conventions and obtaining the official authorization at various political levels. The extent of the study did not allow for clinical diagnosis of PTSD, leaving us with all the limitations and short comings of self-reports.

However, an important limitation is the low response rate of 18%, which makes the generalization and interpretation of the prevalence levels rather difficult. Although the obtained response rate is close to the expected participation of 20% in a postal survey [47], the non-response could be due to three factors. First of all, people may not have been able to respond to the questionnaire because of hospitalization or recovery, physically or emotionally, while also other studies were conducted at the same time. Second, people may not have responded because they felt they were not involved in the disaster, or third, because they were disappointed about the government’s management of the disaster. Finally, it is imaginable that this study may have suffered from response bias. In some cases there may have been an over or under representation due to a lack of independency between the scores of participants belonging to the same family or the same company. The dependency between different participants could possibly have affected the results (e.g. inflating correlations). The design of the study did not allow corrections for this possible dependency.

## Conclusions

This study supports the evidence that the degree of exposure to a disaster, peritraumatic dissociation and the perceived lack of social support determine the development of PTSD symptoms. A second finding of this study is that early development of PTSD symptoms may lead to chronification on the long term. Besides, victims with some posttraumatic stress symptoms in the immediate aftermath of a disaster might appear resilient or adapted to the post-disaster reality, but may be confronted with an exacerbation of their symptoms on the long term (leading to a full PTSD).

Public authorities should invest in pre-disaster research planning and systematic assessment of disaster victims starting with early screening for mental health problems as first phase in a longitudinal design. Research should focus too on the possibilities of on-site psychological assistance to prevent (peritraumatic) dissociative reactions and negative emotions. Foreseeable dissatisfaction with insufficient social support over time may be a target for psychosocial disaster planning too.

## Endnote

^a^Strictly spoken this is not PTSD prevalence given that no clinical assessment has taken place. Here, the term is to be understood as “satisfying all criteria for PTSD”
